# Antibody-mediated diversification of primary and secondary humoral immune responses

**DOI:** 10.1084/jem.20252590

**Published:** 2026-04-28

**Authors:** Dennis Schaefer-Babajew, Laurine Binet, Gabriela S. Silva Santos, Chiara Ruprecht, Lachlan P. Deimel, Mohamed A. ElTanbouly, Dounia Gharrassi, Gabriella Lima dos Reis, Clara Uhe, Kai-Hui Yao, Brianna Hernandez, Parul Agrawal, Anna Gazumyan, Leonidas Stamatatos, Harald Hartweger, Michel C. Nussenzweig

**Affiliations:** 1 Laboratory of Molecular Immunology, The Rockefeller University, New York, NY, USA; 2 Fred Hutchinson Cancer Center, Seattle, WA, USA; 3 Howard Hughes Medical Institute, The Rockefeller University, New York, NY, USA

## Abstract

Humoral immune responses are characterized by increasing antibody affinity and diversity over time. Increased affinity is mediated by a combination of immunoglobulin gene somatic mutation and iterative cycles of selection in germinal centers. Less is understood about how diversity increases. Here, we examine the role of antibody feedback in diversifying immune responses in mice that produce B cells that are incapable of secreting antibodies. To this end, we produced two strains of mice, one that expresses only membrane and secreted forms of IgM, and a second that produces only the membrane-bound form of IgM. Analysis of primary and secondary immune responses shows that antibody feedback significantly diversifies both primary and secondary immune responses even when antibodies are present at levels that are 10–30-fold lower than physiologic. The data have significant implication for sequential vaccination approaches aimed at shepherding immunity to produce broadly neutralizing antibodies to highly diversified pathogens such as HIV-1 and influenza.

## Teaser

Humoral immune responses diversify over time but whether secreted antibodies influence this process is unknown. Using antibody secretion–deficient mice, this study shows a profound impact of secreted antibodies on the evolution of B cell diversity after vaccination.

## Graphical Abstract

**Figure:**
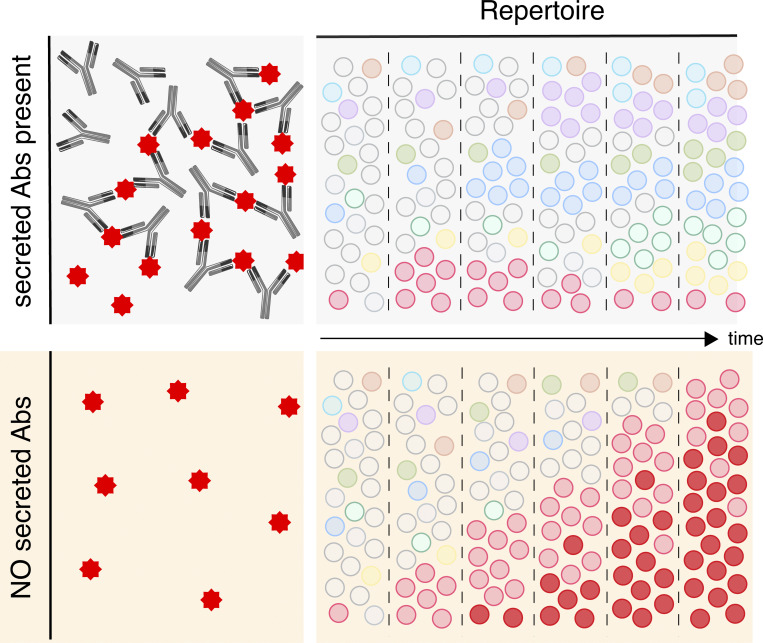

## Introduction

Both the affinity and diversity of antibodies elicited by immunization or infection increase over time by a process that involves iterative cycles of selection, clonal expansion, and somatic mutation in germinal centers (GCs) (Bannard and Cyster, 2017; Victora and Nussenzweig, 2022). These two apparently discordant features of humoral immune responses, increasing affinity and diversity, can be accounted for by dynamic changes in selection over time and by the existence of two cellular compartments, plasma cells (PCs) and memory B cells, respectively. PCs develop by a mechanism that ensures increasing circulating antibody affinity (Zotos and Tarlinton, 2012; Nutt et al., 2015; Kräutler et al., 2017; Ise et al., 2018; Tas et al., 2022; ElTanbouly et al., 2024; MacLean et al., 2025). Memory B cells are selected by a separate mechanism that emphasizes diversity over affinity (Inoue et al., 2021; Inoue et al., 2022; Suan et al., 2017; Viant et al., 2020; Wang et al., 2017).

Antibody diversification becomes increasingly important during secondary or booster immune responses, when only small numbers of high-affinity antigen-binding cells can be detected in GCs (Dal Porto et al., 2002; Eckl-Dorna et al., 2019; Mesin et al., 2019; Malladi et al., 2025). Given the strong selection pressure imposed by iterative cycles of mutation and cell division in the GC, the evolution away from high affinity is counterintuitive. However, diversification may be an important evolutionary adaptation to deal with rapidly changing pathogens such as coronaviruses and influenza virus (Heyman, 2000; Cyster and Wilson, 2024).

Several mechanisms have been suggested to account for the increase in diversity and loss of detectable antigen binding in late primary and secondary GCs. These include the following: (1) continual recruitment of additional lower affinity cells during the reaction (de Carvalho et al., 2023; Hägglöf et al., 2023); (2) increasing T cell fitness that lowers the threshold of B cell selection (Woodruff et al., 2018; Merkenschlager et al., 2023); and (3) antibody-mediated antigen masking or enhancement (Heyman, 2000; Cyster and Wilson, 2024).

The effects of antibody feedback on immune responses have been studied since 1909 when they were first described by Theobald Smith in experiments on diphtheria toxin (Smith, 1909). Recent data show that masking appears to be mediated by high-affinity antibodies and enhancement by low concentrations or low-affinity antibodies that may be present even before immunization or in the early stages of the immune response (Heyman, 2000; Cyster and Wilson, 2024). But because all experiments to date, including Smith’s, have been performed in organisms that have at least some level of circulating antibody, precisely how masking and enhancement contribute to the evolution of humoral immunity and GC dynamics remains to be determined. Understanding these phenomena is increasingly important in developing strategies for sequential vaccination for difficult pathogens such as malaria parasites, HIV-1, and influenza virus where antibody-mediated masking can interfere with the evolution of immunity in response to serial vaccine boosters (Cyster and Wilson, 2024).

Here, we examine the role of antibody feedback in regulating GC responses in mice that have an intact polyclonal B cell compartment but do not secrete antibodies.

## Results

### Antibody secretion–deficient mice

**Figure 1.**
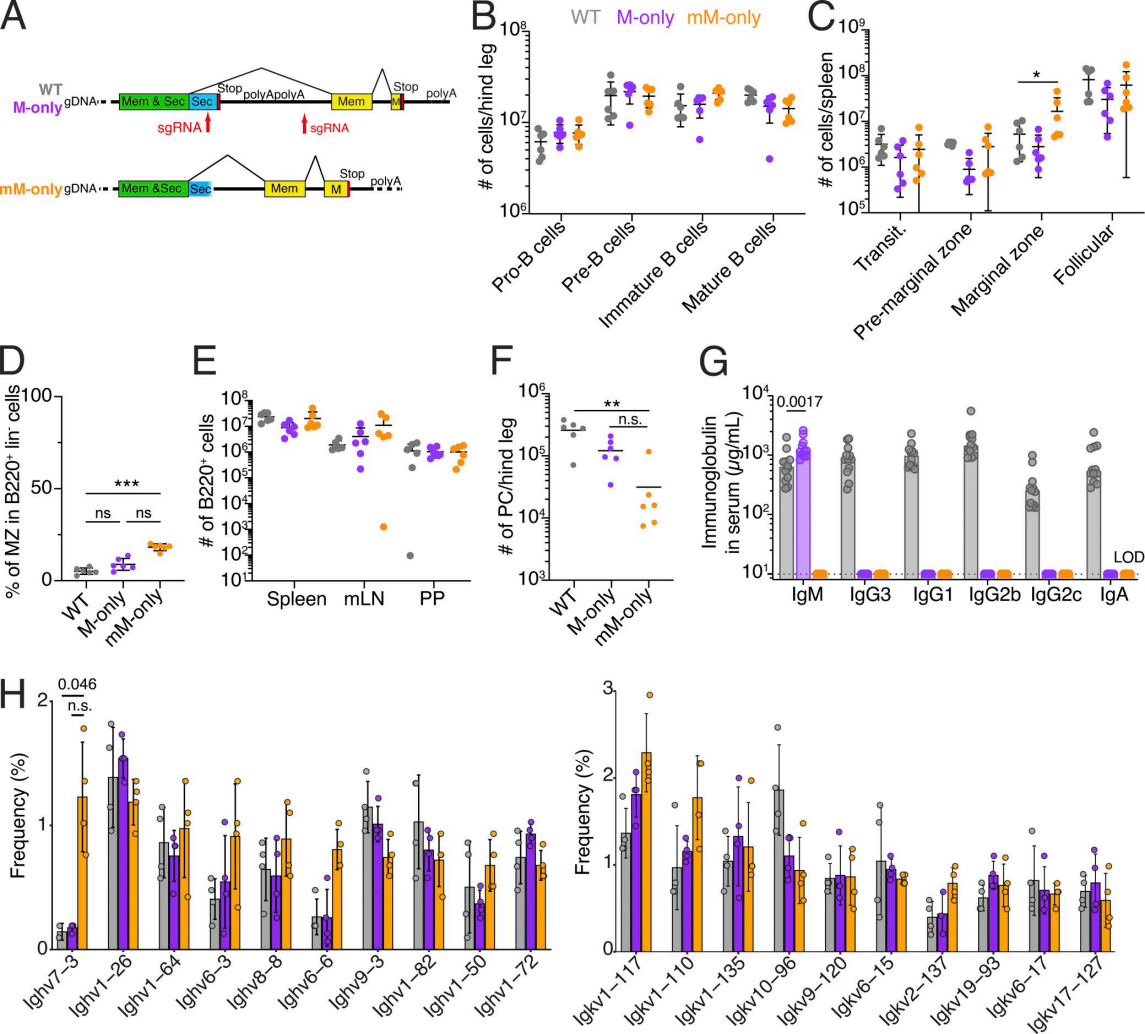
**B cell development in M-only and mM-only mice. (A)** Schematic shows structure of *Ighm* exon 4 and adjacent region in WT, M-only, and mM-only mice. **(B)** Number of pro-, pre-, immature, and mature B cells in the bone marrow on one leg by flow cytometry. Gating as in Fig. S1 A. **(C)** Number of the splenic B cell subsets by flow cytometry. Gating as in Fig. S1 B. **(D)** Percentage of splenic marginal zone B cells. Gating in Fig. S1 B. **(E)** Number of live B cells in spleen, mLN, and PP by flow cytometry. Gating in Fig. S1, B–D. **(F)** Number of PCs in the bone marrow of one leg by flow cytometry. **(G)** ELISA quantification of total IgM, IgG3, IgG1, IgG2b, IgG2c, and IgA in the serum of WT, M-only, and mM-only mice. **(H)** Bar graph depicting the relative abundance of Ighv (left panel) and Igkv (right panel) gene usage in follicular B cells. Top 10 most frequent genes ranked by the mean frequency in mM-only mice are shown. Data were pooled from two independent experiments. Each dot or circle represents a mouse. P values of nonparametric one-way ANOVA with Tukey’s Honest Significant Difference test are shown. All experiments were performed at least twice. *P < 0.05, **P < 0.01, ***P < 0.001. Bars indicate the mean ± SD. PP, Peyer’s patches, LOD, Limit of Detection.

To examine how secreted antibodies impact the development of immune responses, we engineered the IgH locus to produce two strains of mice: a control strain that expresses membrane and secreted forms of IgM (M-only) but no other isotypes; and a second strain whose B cells express only the membrane-bound form of IgM (mM-only) (Fig. 1 A). To this end, the C57BL/6J Ig heavy chain locus was modified using CRISPR-Cas9 to delete a 150-kb region from the 5′ of *Ighd* exon 1 to the 3′ UTR of *Igha*, leaving only the IgM locus intact (M-only mice; see Materials and methods for further details). M-only mice were subsequently engineered to produce the mM-only mice by removing the stop codon of the secreted splice form and its intronic polyadenylation signal in *Ighm* exon 4 (Danner and Leder, 1985; Nussenzweig et al., 1987; Boes et al., 1998; Ehrenstein et al., 1998; Waisman et al., 2007). The later abrogates production of the secreted form by preventing its termination, thereby enforcing splicing to produce the membrane-bound isoform of IgM. We focused on the IgM isotype because it does not bind to most Fc receptors, and therefore, its Fc-mediated activity is generally more limited than other isotypes.

**Figure S1.**
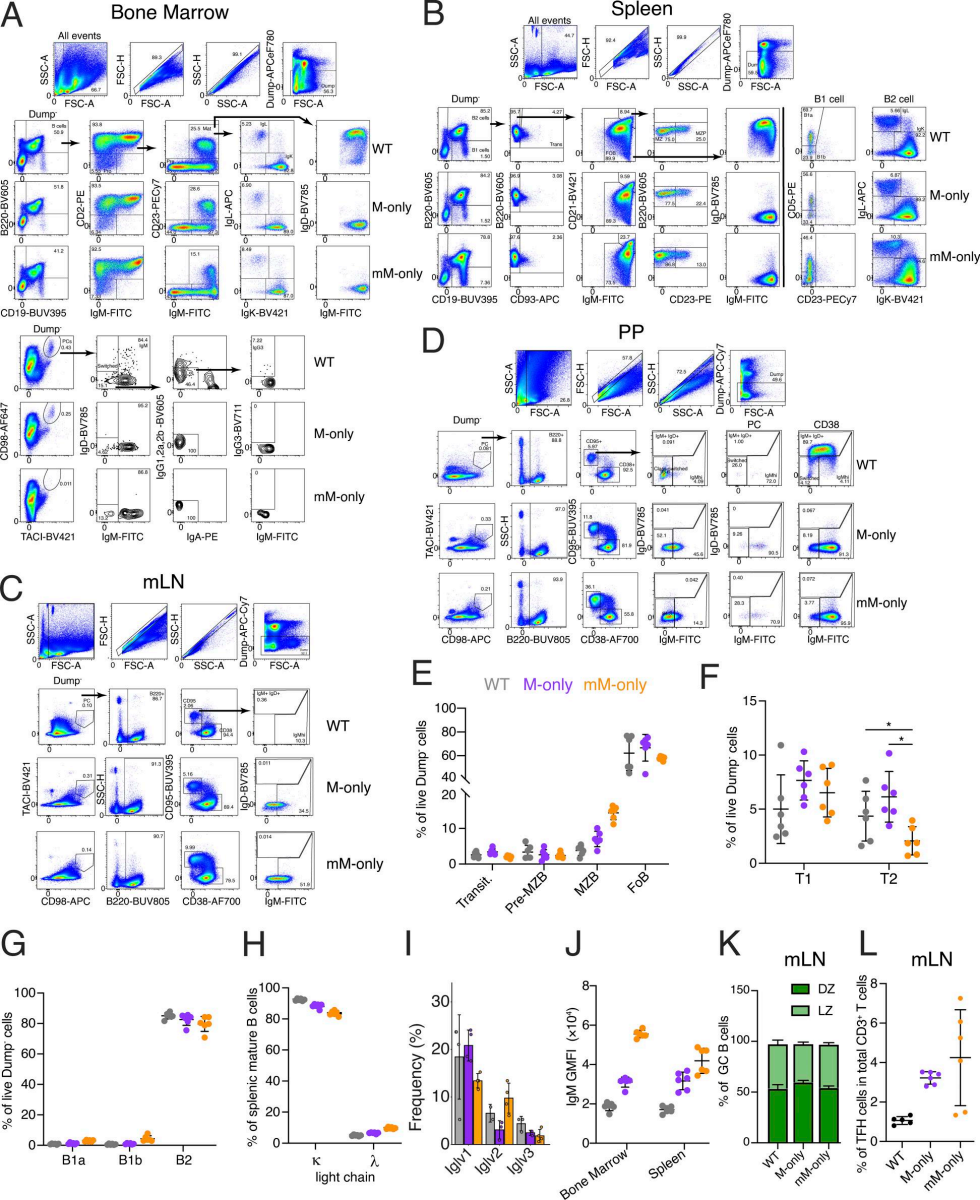
**Analysis of M-only and mM-only mouse strains.** Related to Fig. 1. **(A)** Flow cytometric gating strategy in the bone marrow. All stainings were cell surface and not intracellular stainings. **(B)** Flow cytometric gating strategy in the spleen. **(C)** Flow cytometric gating strategy in the mLN. **(D)** Flow cytometric gating strategy in the PP. **(E)** Percentages of splenic, transitional (Transit), pre-marginal zone (pre-MZB), marginal zone (MZB), and follicular (FoB) cells among live Dump^−^ cells. **(F)** Percentages of transitional T1 and T2 B cells in live Dump^−^ cells. **(G)** Percentages of splenic B1a, B1b, and B2 cells among live Dump^−^ cells. **(H)** Percentages of splenic *κ* or *λ* light chain–bearing mature B cells. **(I)** Bar graphs showing Iglv gene usage in follicular B cells. Top 10 most frequent genes ranked by the mean frequency in mM-only mice are shown. **(J)** GMFI of IgM-FITC in the bone marrow mature B cells and splenic follicular B cells. **(K)** Proportions of dark and light zone B cells in mLNs at steady state. **(L)** Proportions of T follicular helper cells in mLN at steady state in total CD3^+^ T cells. Each dot represents a single mouse. *P > 0.05. Bars indicate the mean ± SD. PP, Peyer’s patches; GMFI, geometric mean fluorescence intensity. DZ, dark zone; LZ, light zone.

Consistent with the observation that membrane-bound IgM regulates B cell development in the bone marrow (Nussenzweig et al., 1987; Kitamura et al., 1991), M-only and mM-only mice showed generally normal numbers of pro-B, pre-B, immature, and mature B cells in the bone marrow (Fig. 1 B and Fig. S1 A). Moreover, the overall numbers of transitional and mature B2 cells in spleen were similar, although a minor decrease in T2 cells was observed in mM-only mice. As reported by others, mice lacking secreted Igs show increases in splenic B1 and marginal zone B cells, but total numbers of B cells were similar in spleen, mesenteric lymph nodes (mLNs), and Peyer’s patches (Fig. 1, C–E; and Fig. S1, B–G) (Hannum et al., 2000; Anderson et al., 2006). *κ*/*λ* light chain ratios showed minor differences in mM-only mice compared with both wild-type (WT) and M-only mice (Fig. S1 H). There were fewer PCs in the bone marrow of M-only mice compared with WT, and they were difficult to detect in mM-only mice (Fig. 1 F and Fig. S1 A). Serum ELISAs performed under steady-state conditions showed slightly increased IgM but no other isotypes in M-only mice and no detectable antibody in mM-only mice (Fig. 1 G, P = 0.0017). Despite the absence of other isotypes, single-cell Ig mRNA sequencing did not reveal any notable differences in Ighv, Igkv, and Iglv usage by follicular B cells between WT, M-only, and mM-only mice except for an enrichment of Ighv7-3 in mM-only mice compared with WT (Fig. 1 H and Fig. S1 I). mM-only B cells showed higher levels of surface IgM expression in both bone marrow and the spleen possibly due to their lack of IgD and inability to secrete (Fig. S1 J). Finally, at steady state, mLN GCs in mM- and M-only mice had the similar dark zone/light zone ratios and number of T follicular helper cells (Fig. S1, K and L). In summary, B cell development appears largely normal, but there are no serum antibodies and few if any PCs in mM-only mice.

### Primary immune responses

**Figure 2.**
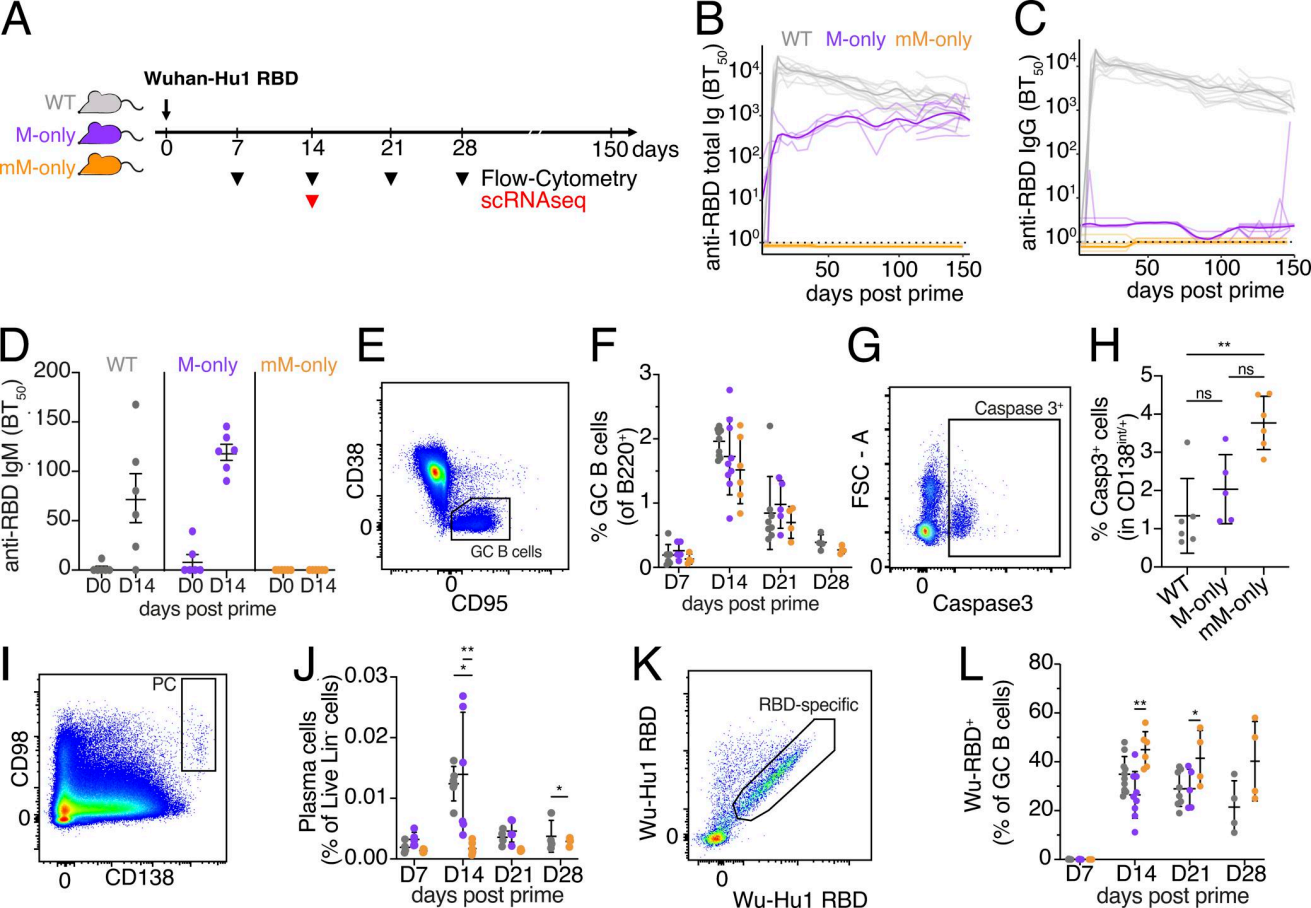
**Primary vaccine responses. (A)** Diagram of the experimental protocol. **(B)** ELISA quantification of anti-RBD antibodies in the serum measured every 3–5 days between day 0 and day 150. **(C)** As in B for IgG anti-RBD antibodies in the serum. Thin lines in B and C represent single mice; bold lines represent group mean. **(D)** Serum anti-RBD IgM ELISA 0 or 14 days after immunization. **(E)** Flow cytometric gating for CD38^−^CD95^+^ GC B cells pregated on B220^+^ cells in dLNs. **(F)** Quantification of GC B cells from E at the indicated time points after immunization. **(G)** Flow cytometric gating for caspase 3^+^ cells among CD138^int/+^ live Dump^−^ cells. **(H)** Quantification of G among CD138^int/+^ live Dump^−^ cells in dLNs 14 days after immunization. **(I)** Flow cytometric gating for CD98^+^ CD138^+^ PC pregated on live Dump^−^ cells in the dLN. **(J)** Quantification of I at the indicated times after immunization. **(K)** Flow cytometric gating of RBD^+^ cells among GC B cells in the dLNs pregated for GC as in E. **(L)** Quantification of K at the indicated time points after immunization. Each dot in all dot plots represents one mouse. P values of nonparametric one-way ANOVA are shown. All experiments were performed at least twice. Additional day 28 data are presented in Fig. S2 B, C, and E. Bars in D, F, H, J, and L indicate the mean ± SD. **P < 0.01, *P < 0.05. dLNs, draining LNs.

To determine how antibodies impact the development of primary B cell immune responses, we immunized mice with the Wuhan-Hu-1 severe acute respiratory syndrome coronavirus 2 (SARS-CoV-2) receptor-binding domain (RBD) (Fig. 2 A). Antigen-specific antibodies were measured by ELISA (Fig. 2, B–D). As expected, M-only mice were limited to the IgM isotype, and mM-only mice produced no serum antibodies in response to the immunization (Fig. 2, B–D). The kinetics of the serological response in M-only mice was similar to WT (Fig. 2, B and D), but the total amount of antibody was lower when considering all isotypes (Fig. 2 B). Notably, whereas the total antibody levels in WT mice decreased over the 150-day period of observation, serum Ig levels were relatively stable and even increased modestly in M-only mice (Fig. 2 B).

**Figure S2.**
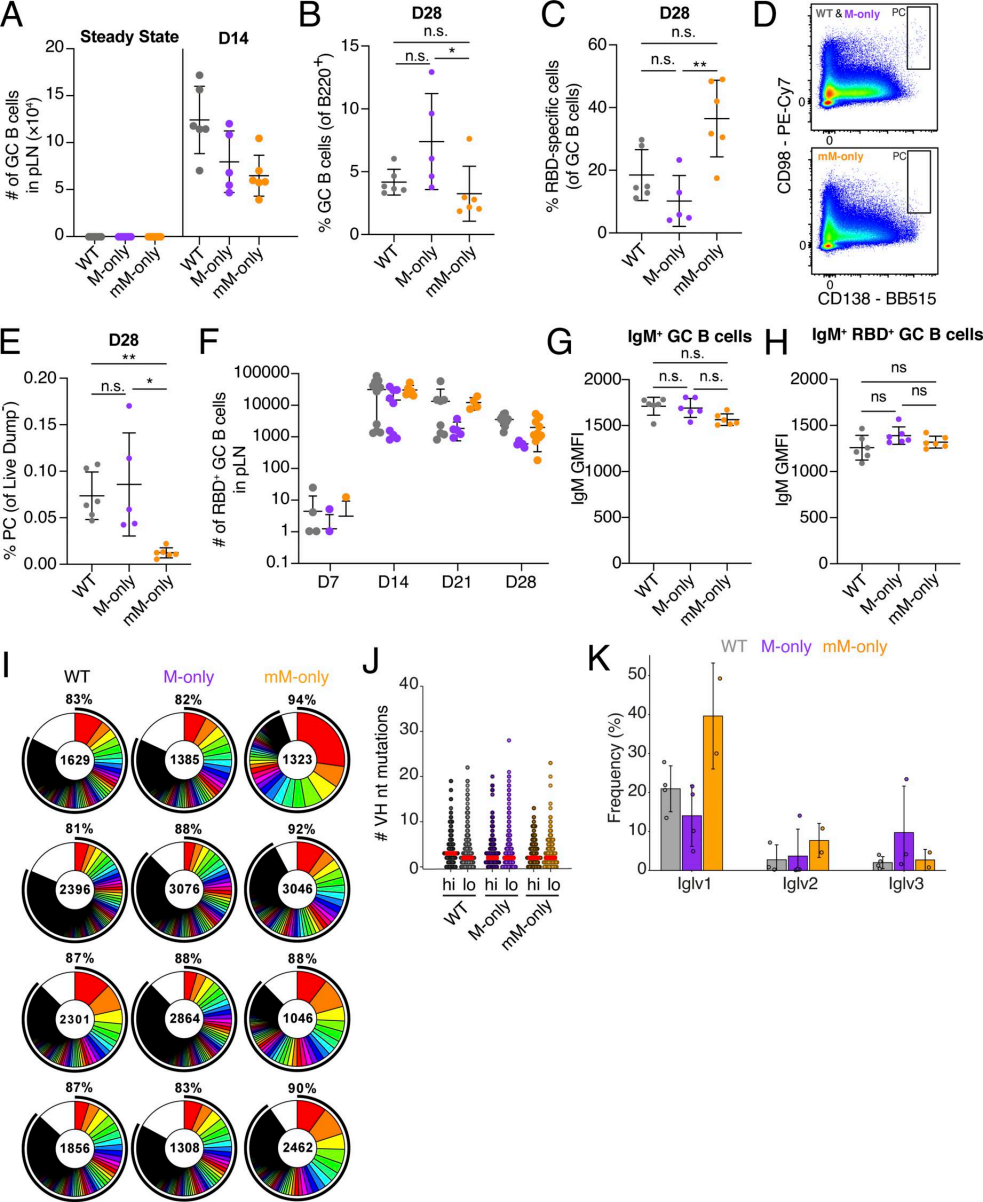
**Prime immunization with RBD.** Related to Figs. 2 and 3. **(A)** Absolute numbers of GC B cells in draining LN at steady state (left) and 14 days after RBD immunization (right). **(B)** Proportions of GC B cells in B220^+^ B cells at day 28 after immunization. **(C)** Proportions of RBD-specific cells in GC B cells at day 28 after immunization. **(D)** Flow cytometric gating strategy for PCs. **(E)** Proportions of PCs in live Dump^−^ cells. **(F)** Numbers of RBD^+^ GC B cells at day 7, day 14, day 21, and day 28 after RBD immunization. **(G)** IgM GMFI in CD95^+^ B220^+^ GC B cells. **(H)** IgM GMFI in RBD-specific CD95^+^ B220^+^ GC B cells. **(I)** Pie charts depicting the distribution of antibody sequences obtained 14 days after prime immunization from GC B cells from four mice/genotype. Inner circle numbers indicate the number of sequences analyzed for each mouse. White section indicates nonexpanded sequences; colored or black pie slices are proportional to the number of clonally related sequences. The outlined black line indicates the percentages of cells in a clonal family. **(J)** Iglv gene usage frequency in genotypes. VH genes split by the mean frequency in mM-only are shown. Top 10 highest frequency (hi) and remaining low-frequency VH genes (lo) are plotted. Each dot represents a VH gene. Bars indicate the median. **(K)** Dot plot showing the number of VH nucleotide somatic hypermutations in sequences from top 10 most frequent V genes in mM-only mice from Fig. 5 F (Hi) compared with sequences from all remaining low-frequency V genes. Each dot indicates VH sequence from one cell. Bars indicate the median. Each dot represents a single mouse. Bars indicate the mean ± SD. *P < 0.05, **P < 0.01 in one-way ANOVA. D, day; GMFI, geometric mean fluorescence intensity.

GC responses were analyzed by flow cytometry on days 0, 7, 14, 21, and 28 after immunization. GCs were absent from popliteal LNs before immunization (Fig. S2 A). The absolute numbers of B cells and the relative proportions of GC B cells among B220^+^ B cells were similar among WT, M-only, and mM-only mice at all time points analyzed (Fig. 2, E and F; and Fig. S2, A–C). Thus, the contribution of antibodies to GC magnitude and kinetics in the primary response to SARS-CoV-2 RBD appears to be limited.

Cells starting to express the PC marker CD138 were evident in LNs of all three strains, but showed increased caspase expression in mM-only mice, and these mice had few detectable mature PCs in LNs or bone marrow (Fig. 1 F, Fig. 2, G–J, Fig. S1 A, and Fig. S2, D and E). The data are consistent with the idea that PCs that are unable to secrete antibody die by apoptosis (Iwakoshi et al., 2003; Todd et al., 2009; Bonaud et al., 2023).

We analyzed the antigen-binding capacity of GC B cells in the primary response by flow cytometry using the Wuhan-Hu-1 RBD labeled with two different fluorophores (Fig. 2, K and L). At the peak of the GC response, 14 days after immunization, there were similar proportions of RBD-binding cells in WT and M-only mice (35% and 26%, respectively), but significantly more in the GCs of mM-only mice (45%, P = 0.0021, Fig. 2 L and Fig. S2 F). Moreover, whereas antigen-binding B cells decreased over time in WT and M-only mice, their relative proportion appeared to persist in mM-only GCs (Fig. 2 L). Differences in B cell receptor (BCR) expression did not account for this difference in antigen binding as IgM surface expression levels were equivalent among genotypes (Fig. S2, G and H). The data are consistent with the idea that secreted antibodies mask immunodominant epitopes and increase antigen valency, thus curbing the relative competitive advantage of high-affinity immunodominant antigen-binding cells in the later stages of the primary GC reaction (Heyman, 2000; Andrews et al., 2015; Schaefer-Babajew et al., 2023; Cyster and Wilson, 2024; Yan et al., 2025).

**Figure 3.**
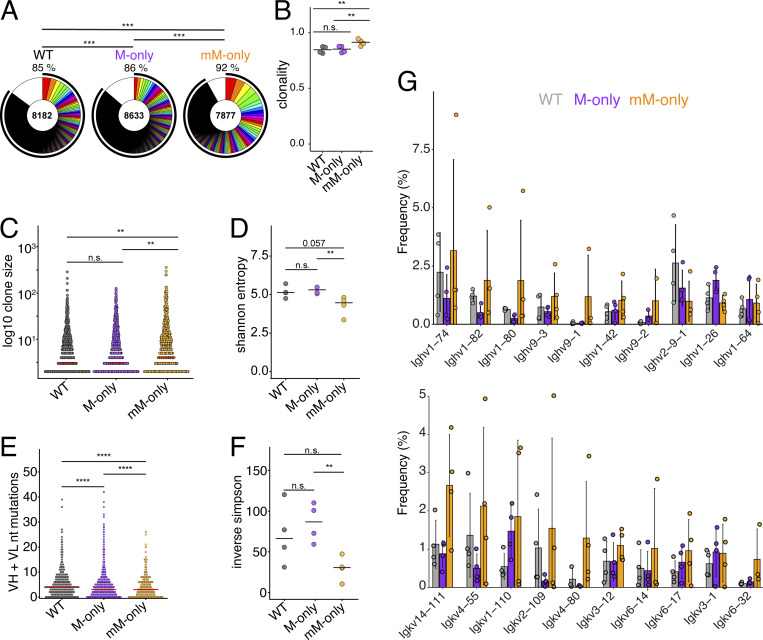
**Primary GC antibody sequences.** **(A)** Pie charts depicting the distribution of antibody sequences pooled from four mice/genotype obtained 14 days after prime immunization from GC B cells. Inner circle numbers indicate the number of sequences analyzed for each genotype. The white section indicates nonexpanded sequences; colored or black pie slices are proportional to the number of clonally related sequences. The outlined black line indicates the percentages of cells in a clonal family. **(B)** Dot plot illustrating the percentage of clonally expanded sequences within each mouse. Each dot indicates one mouse. Bars indicate the mean. **(C)** Dot plot showing clonotype size distribution. Bars indicate the median. Each dot indicates a clone. **(D)** Dot plot showing Shannon’s entropy scores for the sequences depicted in A within each mouse. Bars indicate the median. **(E)** Dot plot showing combined VH + VL gene nucleotide mutations among sequences shown in A. Each dot indicates an antibody VH + VL pair. Bars indicate the median. **(F)** Dot plot depicting inverse Simpson’s index for sequences shown in A for each mouse. Each dot indicates one mouse. Bars indicate the median. **(G)** Bar graphs showing the relative abundance of mouse Ighv and Igkv gene usage among sequences shown in A. Top 10 most frequent genes ranked by the mean frequency in mM-only mice are shown. Bars indicate the mean ± SD. Statistics in A indicate chi-squared test with Monte Carlo P value simulation; P values were subsequently corrected using the Benjamini-Hochberg procedure. Statistics in B–F indicate the two-sided Mann–Whitney U test. ****P < 0.0001, ***P < 0.001, **P < 0.01.

To gain further insights into the effect of antibodies on primary GC responses, we examined clonality, diversity, and IgV gene usage by single-cell VDJ sequencing 14 days after immunization. As expected, expanded clones of B cells were found in GCs of all three genotypes (Fig. 3, A–C and G; and Fig. S2 I). However, the individual clones appeared to be larger and less diverse in the GCs of mM-only mice than in mice that expressed secreted antibodies as evidenced by significantly higher overall clonality and a decrease in the fraction of individual sequences (Fig. 3, B and C). Consistent with this observation, mM-only GC B cells showed reduced Shannon’s and inverse Simpson’s diversity indices, but similar levels of somatic mutation compared with their counterparts, even when analyzing the sequences of clones composed from the top 10 most frequent V genes in mM-only mice (Fig. 3, D–F; and Fig. S2 J). We did not detect significant skewing of IgV gene usage (Fig. 3 G and Fig. S2 K). In conclusion, secreted antibodies appear to be essential for B cell repertoire diversification in primary GC responses to SARS-CoV-2 RBD—a mechanism that appears to be significant even at the early stages of the response.

### Secondary immune responses

**Figure 4.**
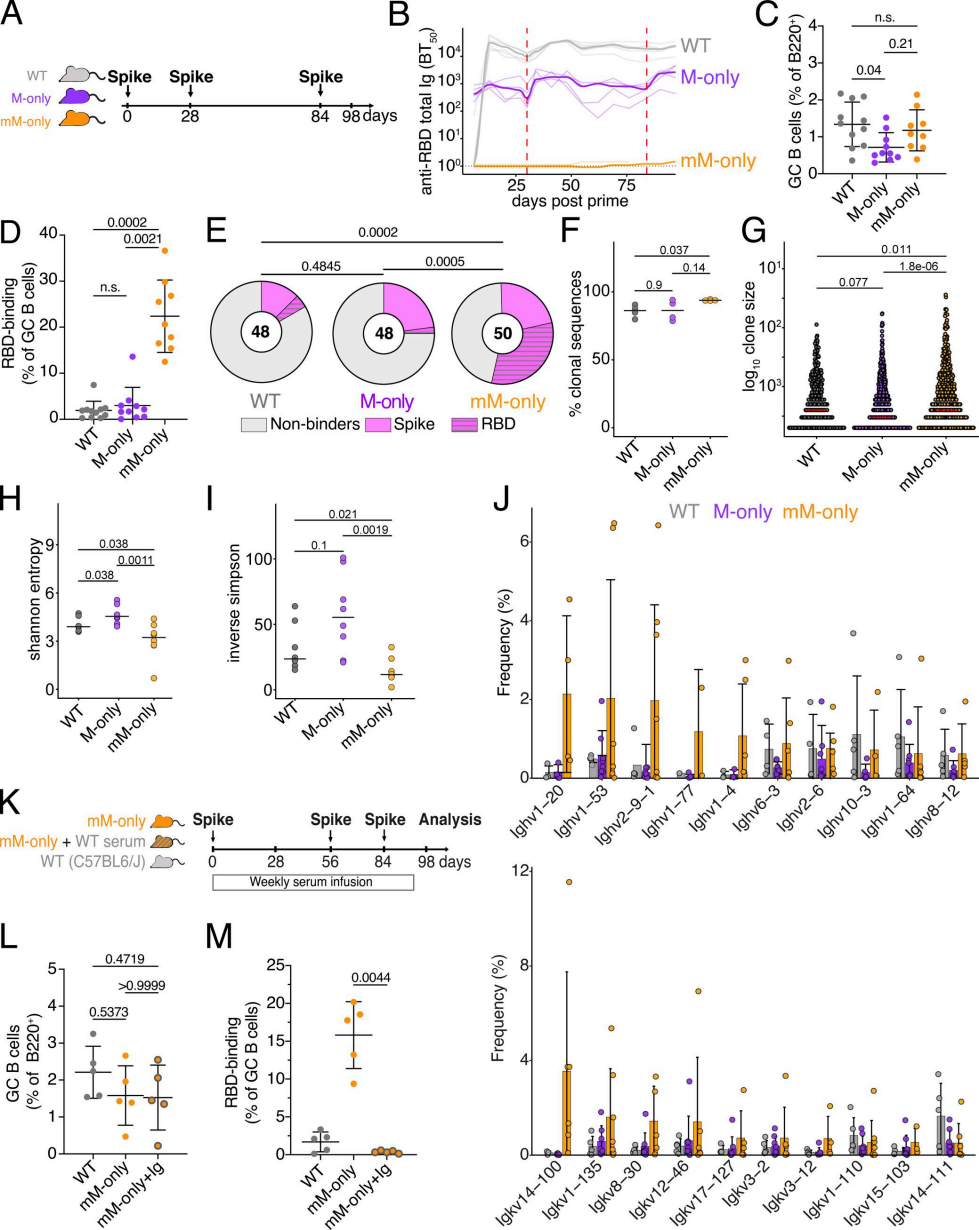
**Prime boost vaccination. (A)** Diagram of the experimental protocol for A–J. **(B)** ELISA quantification of total anti-RBD antibodies in the serum measured weekly from day 6 to day 97 after prime immunization. Thin lines represent single mice; bold line indicates the group mean. Dashed red lines indicate booster immunizations. **(C)** Percentage of GC B cells among B220^+^ B cells in the draining LN at day 98. Gating as in Fig. 2 E. **(D)** Percentage of RBD-binding GC B cells in the draining LN at day 98. Each dot represents one mouse. Gating as in Fig. 2 K. Each dot represents one mouse. Bars indicate the mean ± SD **(E)** Pie charts depicting the distribution of RBD-binding, spike-binding, or nonbinding recombinant antibodies cloned from the largest expanded clones of four mice/group of GC B cells on day 98 from draining LNs. BT_50_ <10 µg/ml is considered binders. Inner circle numbers indicate the number of recombinant antibodies analyzed. **(F)** Dot plot showing the percentage of clonal sequences from GC B cells on day 98. Each dot represents one mouse. Bars indicate the median. **(G)** Number of mutations in GC B cells on day 98. Each dot represents one clone. Bars indicate the median. **(H)** Dot plot depicting Shannon’s entropy for sequences from F. **(I)** Dot plots depicting the inverse Simpson’s index for sequences from F. Each dot represents one mouse. Bars indicate the median. **(J)** Bar graphs showing the relative abundance of mouse Ighv and Igkv gene usage among sequences from F. Top 10 most frequent genes ranked by the mean frequency in mM-only mice are shown. Bars indicate the mean ± SD. **(K)** Diagram of the experimental protocol for L and M. **(L)** Percentage of GC B cells among B220^+^ B cells in the draining LN on day 98. Each dot represents one mouse. Gating as in Fig. 2 E. Bars indicate the mean ± SD. **(M)** Percentage of RBD-binding GC B cells. Each dot represents one mouse. Gating as in Fig. 2 K. Bars indicate the mean ± SD. Statistics in C, D, J, L, and M indicate nonparametric one-way ANOVA P values. Statistics in E–I indicate the two-sided Mann–Whitney U test. n.s., not significant P > 0.05. All flow cytometric experiments are from at least two independent experiments.

To determine whether antibodies developing during the primary response influence subsequent booster responses, we vaccinated WT, M-only, and mM-only mice in accordance with protocols used during human immunization against SARS-CoV-2 using the full-length–stabilized SARS-CoV-2 spike protein (Fig. 4 A).

**Figure S3.**
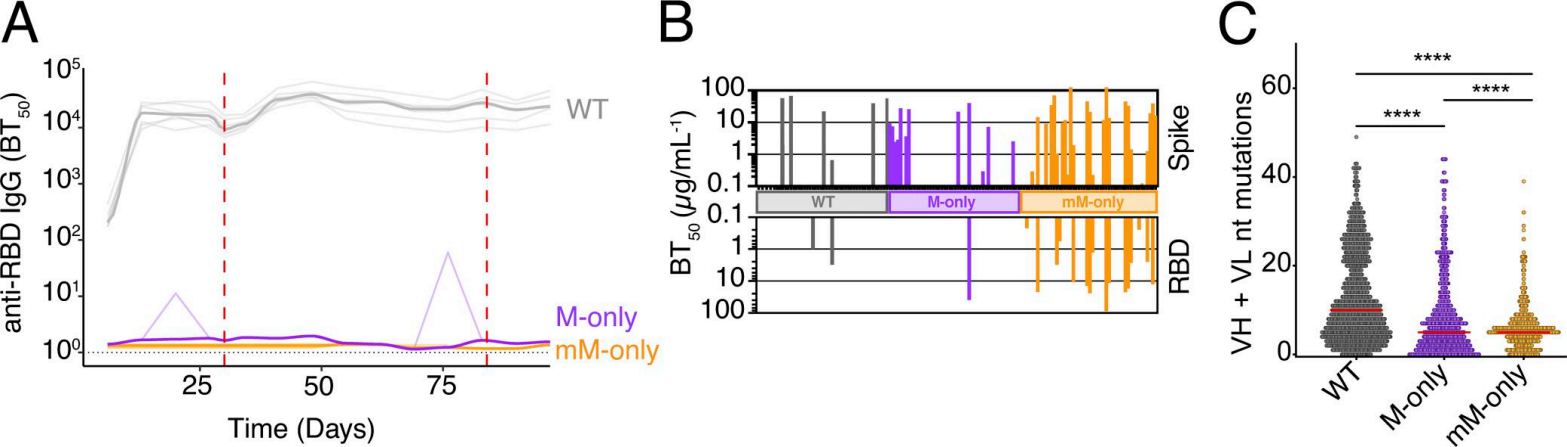
**Analysis of anti-spike prime boost responses.** Related to Fig. 4. **(A)** ELISA quantification of anti-RBD IgG antibodies in the serum of mice from Fig. 4 measured weekly from day 6 to day 97. Thin lines represent single mice; bold lines represent the group mean. **(B)** Bar graph of recombinant antibodies binding to spike (top) or RBD (bottom) protein. Each bar represents one antibody. **(C)** Dot plot depicting somatic hypermutations in GC B cells on day 98 from Fig. 4. Each dot indicates one sequence. Bars indicate the median. P value of the two-sided Mann–Whitney U test is indicated. ****P < 0.0001.

Serum antibody responses were measured by ELISA at approximately weekly intervals from days 6 to 97 after immunization. As in the primary responses, antibodies were not detectable in mM-only mice (Fig. 4 B and Fig. S3 A), and M-only mice produced ∼30-fold less serum antibody compared with WT mice 14 days after boosting. Flow cytometry on cells obtained from draining LNs showed that M-only had lower proportions of GC B cells, but mM-only was not significantly different from WT (Fig. 4 C). Thus, the complete absence of secreted antibodies does not impact the ability to form or sustain secondary GCs.

Antigen-binding GC B cells were enumerated using fluorescently labeled Wuhan-Hu-1 RBD since this is the primary target of the anti-spike immune response in mice and humans. Notably, the three strains differed significantly in the fraction of Wuhan-Hu-1 RBD-binding B cells (Fig. 4 D). Whereas only 1.9% and 3% of the B cells in WT or M-only GCs bound to RBD, 22% of mM-only GC B cells showed demonstrable binding (P = 0.002, Fig. 4 D). These results are consistent with the finding that partial antibody depletion in contralaterally boosted mice resulted in an increase in antigen-binding cells in GCs (Schiepers et al., 2024).

To determine the antigen-binding specificity of GC B cells that develop after boosting, we cloned and expressed 146 representative antibodies and tested them for binding to Wuhan-Hu-1 spike and the isolated RBD by ELISA (Table S1). Altogether, only 17% (WT) and 25% (M-only) of mAbs isolated from secondary GCs exposed to endogenous antibodies showed measurable binding to the immunizing antigen, whereas 54% of the mAbs isolated from mM-only GCs were of sufficiently high affinity to bind by ELISA (Fig. 4 E and Fig. S3 B). Whereas 32% of antibodies obtained from mM-only mice bound to the immunodominant RBD, only 4.2% and 2.1% of mAbs from WT and M-only GCs did so, respectively (P = 0.0002 and 0.0005, respectively, Fig. 4 E). The fraction of antibodies binding to non-RBD epitopes on the spike was 12.5%, 23%, and 22% in WT, M-only, and mM-only mice, respectively. To examine the nature of the antibodies produced after booster immunization, we compared the Ig sequences from single GC B cells. GC B cells obtained from secondary GCs of mM-only mice were more clonal and significantly less diverse than those obtained from M-only mice (Fig. 4, F–I). Again, we did not detect significant differences in V gene usage and genotypes displayed similar numbers of somatic mutations (Fig. 4 J and Fig. S3 C). The data suggest that antibody feedback inhibits accumulation of GC B cells that are restricted to the initial immunodominant epitope even when those antibodies are restricted to the IgM isotype and when total Ig concentrations are 30-fold lower than physiologic.

**Figure S4.**
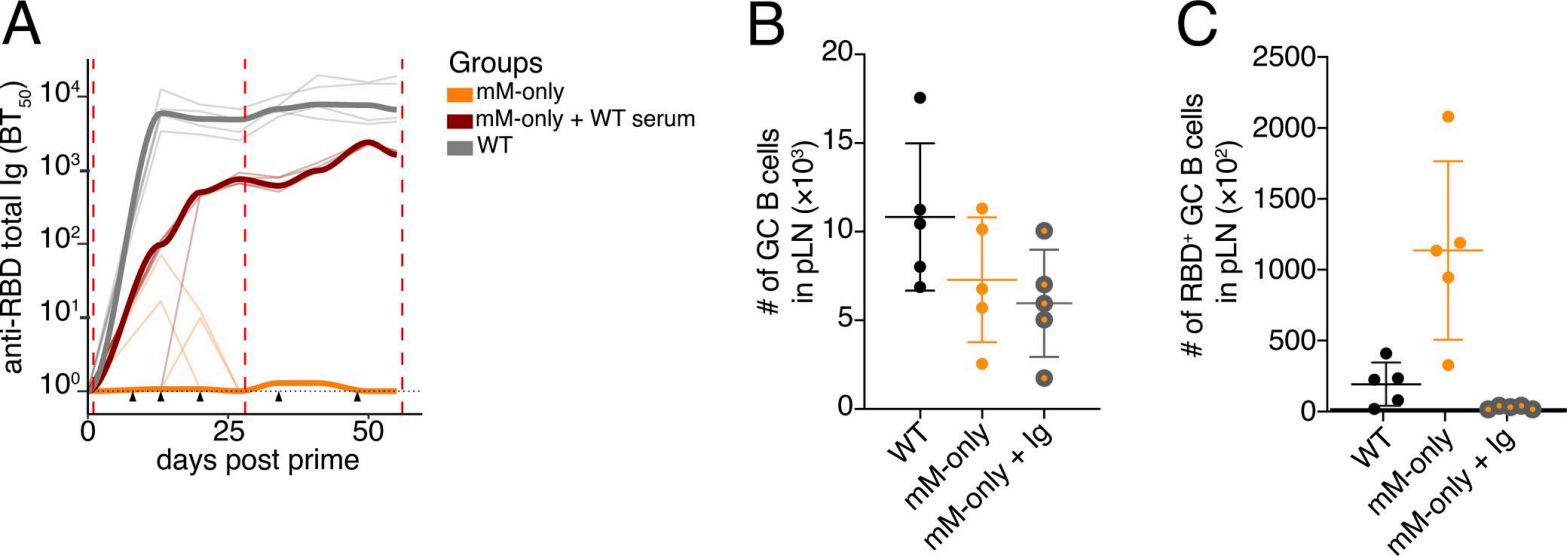
**Analysis of serum transfer experiment.** Related to Fig. 5. **(A)** ELISA quantification of anti-RBD total Ig antibodies in the serum measured weekly from day 0 to day 55. Red dashed lines indicate immunizations; black triangles indicate serum transfer. Thin lines indicate individual mice; bold lines indicate the group mean. **(B)** Number of GC B cells in the dLN. **(C)** Number of RBD-specific GC B cells in the dLN. Each dot represents a single mouse; bars indicate the mean ± SD. dLN, draining LN.

To determine whether passively transferred antibodies are sufficient to revert the mM-only phenotype, we repeated the prime boost experiment with Wuhan-Hu-1 spike and transferred contemporaneous serum from WT mice into mM-only recipients (Fig. 4 K). As determined by ELISA, serologic reconstitution was only partial with an ∼10-fold lower level of specific antibodies in the mM-only recipients compared with their WT counterparts (Fig. S4 A). Notably, while the fraction of GC B cells in draining LNs was similar between genotypes, passive antibody transfer completely reverted the mM-only phenotype resulting in near-complete loss of Wuhan-Hu-1 RBD-binding B cells in the mM-only GCs (Fig. 4, L and M; and Fig. S4, B and C). We conclude that small amounts of polyclonal antigen-specific serum are sufficient to drastically change antigen-binding specificity in the GC. This observation is consistent with the idea that secreted antibodies mask immunodominant epitopes and thereby diversify epitope usage in GCs during polyclonal immune responses.

**Figure 5.**
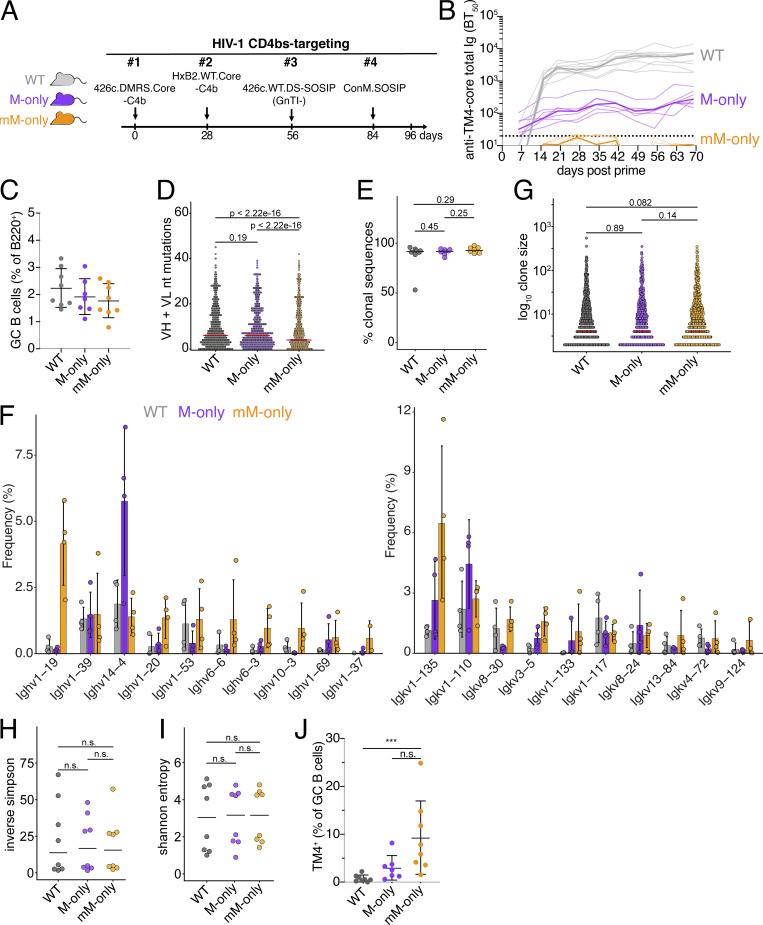
**Sequential immunization with an HIV-1 antigen series. (A)** Diagram of the experimental protocol. **(B)** ELISA quantification of total anti-TM4 core serum antibodies measured weekly from days 6 to 69. Thin lines represent single mice; bold lines represent the group mean. **(C)** Percentage of GC B cells among B220^+^ B cells in the draining LN on day 96. Gating as in Fig. 2 E. Bars indicate the mean ± SD. **(D)** Dot plot showing combined VH + VL gene nucleotide mutations among GC B cell sequences on day 96. Each dot indicates an antibody VH + VL pair. Bars indicate the median. **(E)** Proportions of clonal sequences within each mouse at day 96. Each dot indicates sequences from one mouse. Bars indicate the median. **(F)** Bar graph showing the relative abundance of mouse Ighv (left panel) and Igkv (right panel) gene usage in GC B cells on day 96. Top 10 most frequent genes ranked by the mean frequency in mM-only mice are shown. Bars indicate the mean ± SD. **(G)** Dot plot depicting the percentage of clonal sequences from GC B cells on day 96. Each dot indicates a clone. Bars indicate the median. **(H)** Dot plot depicting inverse Simpson’s index of antibody sequences from day 96 GC B cells. Each dot represents one mouse. Bars indicate the median. **(I)** Dot plot showing Shannon’s entropy index from day 96 GC B cells. Each dot represents one mouse. Bars indicate the median. **(J)** Percentages of TM4-binding GC B cells. P values of nonparametric one-way ANOVA are indicated. ***P < 0.001.

Sequential immunization strategies designed to produce broadly neutralizing antibodies (bNAbs) to HIV-1 and influenza virus are currently being investigated in animal models and in the clinic. The multistep vaccine concept being tested involves using mutated, high-affinity priming immunogens to recruit rare bNAb precursors followed by iterative administration of progressively more native antigens to shepherd B cell responses through a series of somatic mutations required to produce bNAbs (Escolano et al., 2016; Burton, 2019; Haynes et al., 2023). At the end of the sequence, antigen-binding B cells should retain binding to the priming immunogen and in addition bind to all the different antigens used in the vaccine. This idea has yet to produce necessary broadly protective serologic responses in part because “off-target” antibodies to alternative sites on the immunogen dominate the response after booster vaccination (Escolano et al., 2019; Escolano et al., 2021). To determine how circulating antibodies might contribute to this effect, we immunized mice with a series of four HIV-1 CD4-binding site–targeting immunogens, the first of which is currently in early clinical testing (NCT05471076; 426c.DMRS.Core [TM4] antigen, Fig. 5 A) (McGuire et al., 2016). Immunizations were administered in the same anatomic location throughout, and longitudinal ELISA analysis for antibodies binding to 426c.DMRS.Core showed that titers were present throughout the immunization protocol in WT and M-only but not mM-only mice (Fig. 5 B).

**Figure S5.**
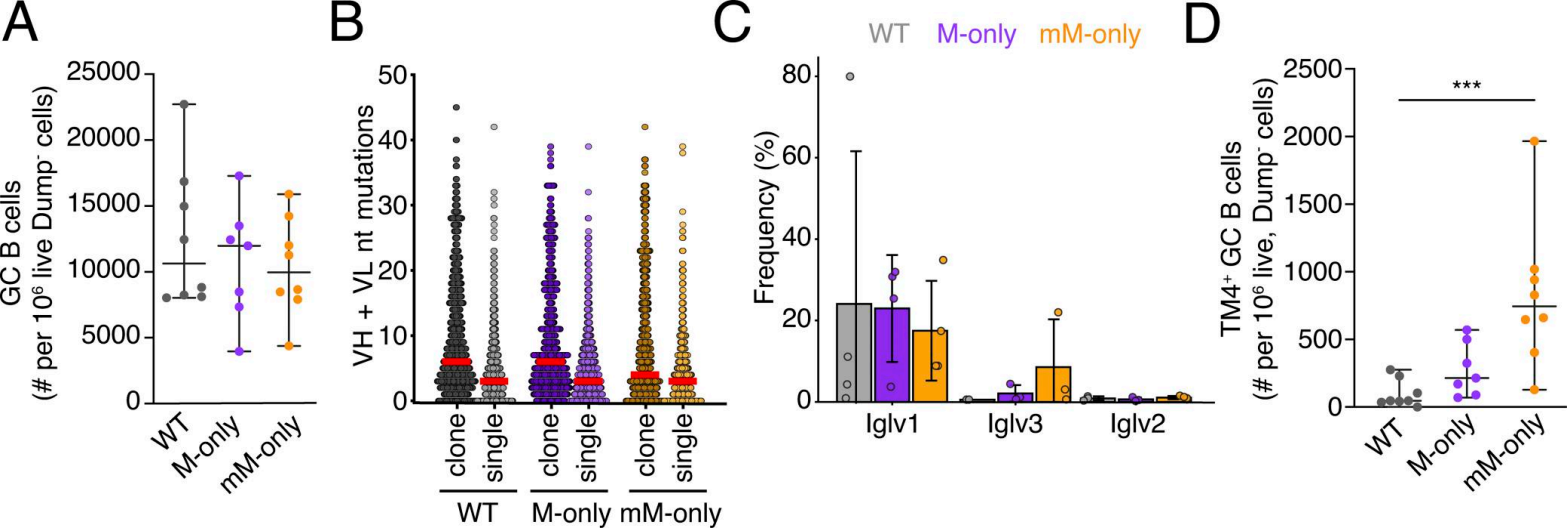
**Analysis of anti-HIV-1 sequential immunization.** Related to Fig. 5. **(A)** Number of GC B cells in draining LNs after sequential immunizations on day 96. **(B)** Dot plot showing combined VH + VL gene nucleotide mutations among clonally expanded sequences compared with nonexpanded sequences (singlets). Each dot indicates an antibody VH + VL pair. Bars indicate the median. **(C)** Bar graphs showing Iglv gene usage in GC B cells. Top 10 most frequent genes ranked by the mean frequency in mM-only mice are shown. **(D)** Number of TM4-binding GC B cells in sequentially immunized mice on day 96 in draining LNs. Each dot represents a single mouse; bars indicate the mean ± SD unless otherwise indicated. ***P < 0.001; nonparametric one-way ANOVA P values are shown.

14 days after the last boost, GC B cell numbers were equivalent among the three mouse strains with median GC B cell frequencies between 1.7% and 2.2% of all B cells in the draining LN (Fig. 5 C and Fig. S5 A). Somatic hypermutation was again comparable across genotypes including expanded clones (Fig. 5 D and Fig. S5 B). Clonality, clone size, IgV gene usage, and measures of diversity did not show significant differences (Fig. 5, E–I; and Fig. S5 C), and considering all cells irrespective of HIV-1 antigen binding, we saw a high degree of clonality in all three groups (Fig. 5 E). Antigen binding to TM4, but not to other sequentially delivered antigens, was measured by flow cytometry. Notably, at the end of the sequential vaccine sequence WT, M-only, and mM-only showed 0.76%, 3%, and 9% TM4-binding GC cells, respectively (Fig. 5 J and Fig. S5 D). Thus, only mM-only mice that are devoid of circulating antibodies show continued levels of GC B cells that bind to the priming immunogen after a sequential series of HIV-1 vaccine boosters. In summary, antibody feedback interfered strongly with persistence of antigen-binding GC B cells in sequential immunization, but differences in clonality and diversity were less pronounced compared with repeated SARS-CoV-2 spike protein immunization. We speculate that this difference arises partly from the nature of the antigens and reduced antibody feedback associated with the sequential use of different antigens. We conclude that circulating antibodies interfere with GC persistence of B cells that retain the ability to bind to the priming immunogen.

## Discussion

GCs are microanatomic compartments specialized for affinity maturation and diversification of humoral immune responses. Affinity maturation is an iterative process involving repetitive cycles of division and mutation in the GC dark zone followed by migration to the light zone where B cells test their newly acquired antigen receptors for binding and capture of antigen deposited in follicular dendritic cells. Selection is mediated by a combination of BCR signaling and T follicular help (Victora and Nussenzweig, 2022; Chen et al., 2023). How GCs diversify antibody responses in the face of strong positive selection is less well understood. Our data indicate that even small amounts of specific polyclonal antibodies produced during the immune response are sufficient to diversify the GC. We hypothesize that this phenomenon is due to masking responses to immunodominant epitopes and producing immune complexes that lower the affinity thresholds for B cell entry into the GC.

Antibody feedback has been studied primarily by passive antibody transfer in animals and humans (Heyman, 2000; Schaefer-Babajew et al., 2023; Cyster and Wilson, 2024). These experiments revealed that both IgM and IgG can produce immune complexes and mediate masking through interactions with Fc and complement receptors (Heyman, 2000). Moreover, recent work in antibody knock-in mice producing antibodies of differing affinity to a selected epitope showed that the antibody feedback effects were dependent on affinity and that changes in the T cell compartment might also contribute (Barbulescu et al., 2025; Yan et al., 2025). In addition, even partial deletion of antibody-secreting cells showed that masking impacts the composition of the GC (Schiepers et al., 2024). Our experiments extend these findings to primary and secondary immune responses in animals that have an otherwise intact polyclonal B and T cell compartment but are unable to produce secreted antibodies. Our data show that antibody feedback is a major mechanism for diversification of both primary and secondary humoral immune responses.

Diversification by antibody feedback has the potential to enable recall responses to rapidly evolving pathogens. For example, although initial responses to SARS-CoV-2 were focused on highly strain-specific epitopes, antibody feedback diversified the response to include more conserved epitopes that offered some protection against newly arising variants. However, our data indicate that these feedback effects pose a very significant barrier to sequential immunization approaches such as those being tested for HIV-1 that aim to focus immunity to a singular broadly neutralizing epitope.

## Materials and methods

### Mice

C57BL/6J mice were purchased from Jackson Laboratories. M-only and mM-only mice were created and maintained at the Rockefeller University with assistance from the Rockefeller University CRISPR and Genome Editing Center and Transgenic and Reproductive Technology Center, New York, NY, USA. Cas9 (IDT) and single guide RNA (sgRNA) (IDT) ribonucleoprotein complexes targeting the IgH locus were electroporated into C57BL/6J single-cell mouse embryos, which were then recovered and incubated overnight at 37 °C before implantation into female foster mother mice. For M-only mice, sgRNA spacer sequences 5′-GTA​GAT​CTC​TTC​CTA​AGA​GG-3′ and 5′-TTA​CTA​GGC​TCC​TCC​ATA​TG-3′ were used to delete *Ighd*-*Igha*. M-only mice were retargeted with sgRNAs with spacer sequences 5′-CGC​CTG​TGT​CAG​ACA​TGA​TC-3′ and 5′-GGG​TAG​GAC​AAG​CAA​CGC​AC-3′ to delete the part of *Ighm* exon 4 only found in the secreted IgM splice form, as well as the subsequent stop codon and alternative, intronic polyadenylation site (Danner and Leder, 1985; Boes et al., 1998; Ehrenstein et al., 1998; Waisman et al., 2007). Cutsite adjacent deletions were verified by PCR from tail DNA and Sanger sequencing of PCR products. Sequences were analyzed using Geneious Prime (GraphPad). Mice were bred to homozygosity. The presence or absence of antibody isotypes was verified by flow cytometry and ELISA of serum in homozygous animals from homozygous parents due to maternal transfer of antibodies in utero and through suckling.

Male and female mice aged 6–12 wk were used. Animals were housed at the Rockefeller University Comparative Bioscience Center, and all animal procedures were performed following protocols approved by the Rockefeller University Institutional Animal Care and Use Committee. Animals were housed at an ambient temperature of 22°C and a humidity of 30–70% under a 12–12-h light–dark cycle with free access to food and water. For experimental endpoints, mice were euthanized using CO_2_, followed by cervical dislocation.

### Immunization

All immunizations were performed by subcutaneous footpad injection of 5 µg of immunogen in 33% alhydrogel (InvivoGen).

### Serum infusion

Serum was obtained from immunized, time-matched C57BL/6J mice and pooled, before being administered to recipients at the equivalent time points. Each recipient received 200 μl of the pooled serum on a weekly basis.

### Flow cytometry

For steady-state experiments, mice aged from 10 to 11 wk were sacrificed, and their spleen, mLN, Peyer’s patches, and bone marrow were isolated and dissociated to obtain single-cell suspensions. Bone marrow from pelvic bone, femur, and tibia was isolated by centrifugation at 10,000 *g* for 15 s. Spleen and Peyer’s patches were forced through a 70-µm cell strainer. LNs were collected into 1.5-ml Eppendorf tubes and dissociated using a pestle. Spleen and bone marrow pellets were then incubated in ACK lysis buffer for 15 min on ice, washed, and processed together with the LN as described below.

For all experiments, single-cell suspensions were incubated in Fc block (BD Biosciences) for 15 min. Cells were then centrifuged at 350 *g* for 5 min and resuspended and incubated in a solution of PBS-diluted fluorescent–biotin–antigen tetramers for 30 min on ice. Additional labeled antibodies were then added to the cells, and samples were incubated for an additional 30 min on ice. For intracellular staining of caspase 3, cells were permeabilized using the eBioscience Foxp3 permeabilization kit (reference: 00-5523-00), washed in the supplied permeabilization buffer, and stained for 30 min at 4°C as per the manufacturer’s instructions. The different cell populations were identified as follows: pro-B (Dump^−^, CD19^+^, B220^+^, IgM^−^, CD2^−^), pre-B (Dump^−^, CD19^+^, B220^+^, IgM^−^, CD2^+^), immature B (Dump^−^, CD19^+^, B220^+^, IgM^+^, CD2^+^, CD23^−^), mature B (Dump^−^, CD19^+^, B220^+^, IgM^+^, CD2^+^, CD23^+^), B1a (Dump^−^, CD19^+^, B220^−^, CD23^−^, CD5^+^), B1b (Dump^−^, CD19^+^, B220^−^, CD23^−^, CD5^−^), B2 (Dump^−^, CD19^+^, B220^+^), transitional B (Dump^−^, CD19^+^, B220^+^, CD93^+^), pre-marginal zone B (Dump^−^, CD19^+^, B220^+^, CD21^hi^, CD93^−^, IgM^hi^, CD23^+^), marginal zone B (Dump^−^, CD19^+^, B220^+^, CD21^hi^, CD93^−^, IgM^hi^, CD23^−^), follicular B (Dump^−^, CD19^+^, B220^+^, CD21^int^, CD93^−^, IgM^+^), PCs at steady state (Dump^−^, CD98^+^, TACI^+^) or after immunization (Dump^−^ CD98^+^ CD138^+^), and GC B cells (Dump^−^, CD98^−^, CD138^−^, B220^+^, CD95^+^, CD38^−^). The Dump channel contains Ly6G, F4/80, NK1.1, CD4, and CD8 antibodies and Zombie-NIR live/dead marker. Flow cytometric reagents are listed in Table S2.

Antigen tetramer staining was performed with a combination of two different fluorescently labeled streptavidin conjugates per antigen. Biotinylated antigens at 5 µg/ml were individually preincubated with each streptavidin–fluorophore (all from BioLegend) at a 1 µg/ml dilution before staining to allow tetramer formation, then combined, and cells were resuspended in the mixture and incubated for 30 min on ice, before staining for other surface markers. Samples were acquired on a BD LSRFortessa or BD Symphony A3 or A5. All cell sorting was performed on BD FACSymphony S6. Data were analyzed using FlowJo version 10.10.0.

### Recombinant protein production

All expression vectors for recombinant protein production were confirmed by Sanger (Azenta) or Oxford Nanopore (Plasmidsaurus) sequencing. The construct encoding the RBD of SARS-CoV-2 (GenBank MN985325.1; S protein residues 319–539) was previously described (Barnes et al., 2020). All recombinant proteins were produced in Expi293F cells (A14527; Gibco), except 426c.WT.SOSIP, which was produced in Expi293F GnTI- cells (A39240; Gibco). Transient transfections of expression plasmids were performed using the ExpiFectamine 293 transfection kit (A14525; Gibco) as previously described (McGuire et al., 2014). In brief, 4–6 days after transfection, the culture supernatant was harvested, centrifuged to pellet cells, and sterilized by filtration for affinity chromatography. Trimeric Env proteins were purified by passing the supernatant through an agarose-bound *Galanthus nivalis* lectin resin (Vector Laboratories) and subsequent size-exclusion chromatography. A Ni Sepharose 6 Fast Flow resin (117531803; Cytiva) was used for purification of His-tagged proteins. Native gel electrophoresis identified peak fractions from size-exclusion chromatography. Fractions corresponding to monomeric Envelope trimers, spike S6P protein trimers, or RBD monomers were pooled and stored at −20°C. Antibodies were purified over a protein G Sepharose 4 Fast Flow resin (70611805; Cytiva) and buffer-exchanged into PBS and stored at −80°C.

For random biotinylation, proteins were biotinylated using the EZ-Link Sulfo-NHS-LC-Biotinylation kit according to the manufacturer’s instructions (31497; Thermo Fisher Scientific). Excess biotin was removed by diafiltration with 100 kDa cutoff. The biotinylated protein was stored at −20°C or −80°C.

Avi-tagged TM4 core gp120 or RBD was biotinylated using a BirA reaction according to the manufacturer’s instructions using a fivefold molar excess of biotin (EC6.3.4.15; Avidity), buffer-exchanged to PBS, and stored at −80°C.

### ELISA

All ELISAs used Costar 96-well, half-area, high-binding, polystyrene assay plates (Cat.# 3960; Corning), which were coated with the indicated antigen (TM4, SARS-CoV-2 Wuhan-Hu-1 RBD or spike protein, all produced in-house) or anti-isotype antibody (anti-IgM, 1020-01; Southern Biotech; anti-IgG3, 1100-01; Southern Biotech; anti-IgG1, 1070-01; Southern Biotech; anti-IgG2b, 1090-01; Southern Biotech; anti-IgG2c, 115-005-208; Jackson ImmunoResearch; anti-IgA, 1040-01; Southern Biotech) at 2–5 µg/ml in PBS overnight at 4°C. Plates were blocked with 5% skimmed milk powder in PBS or 1% BSA, 0.1 mM EDTA, 0.05% Tween-20 in PBS for 2 h at room temperature. Six washes in PBS/0.05% Tween-20 were performed after every subsequent step. Sera were diluted in PBS at 1:50–1:100 top dilution and 1:3 (naive and after prime) or 1:4 (after boosts) or 1:5 (steady-state serum antibody levels) serially diluted for an 8-point curve. Isotype antibody standards (5300-01B; Southern Biotech) were diluted to 10 µg/ml and diluted 1:5 in PBS for an eight-point curve. Secondary antibodies conjugated to horseradish peroxidase were used to detect bound antibodies mouse IgG (1:5,000, Cat.# 115-035-071; Jackson ImmunoResearch or Cat.# 1030-05; Southern Biotech), or mouse IgM, IgG3, IgG1, IgG2b, or IgA (Cat.# 5300-05B; Southern Biotech), or IgG2c (115-035-208; Jackson ImmunoResearch), or total Ig (anti-kappa combined with anti-lambda light chain, Cat.# 5300-05B; Southern Biotech). HRP substrate 3,3′,5,5′-tetramethylbenzidine (Cat.# 34021; Thermo Fisher Scientific) was used for development, and the reaction was stopped by adding an equal volume of 1 M H_2_SO_4_ (Sigma-Aldrich). Absorbance was read at 450 and 570 nm on a FLUOstar Omega (BMG LABTECH). For analysis of steady-state antibody concentration in serum, isotype standards were included on every plate and used to fit a sigmoidal 4-parameter logistic regression standard curve in GraphPad Prism 10, which was used to interpolate serum concentrations from dilutions in the exponential phase of the curve. For total anti-antigen responses, sigmoidal 4-parameter logistic regression was fitted to curves of every sample and used to calculate the half-maximal binding titer (BT_50_). µg/ml values below detection were set to the minimum detection level based on control sera to avoid plotting 0 on logarithmic plots. For samples where the signal was too low for a curve fit, BT_50_ was set to 10, which is 5× above the highest measured dilution.

### 10X Genomics and single-cell libraries

Indicated mice were immunized with either spike, RBD, or HIV proteins. Cell suspensions were prepared as mentioned before from popliteal LNs, and samples were indexed using TotalSeq-C cell surface antibodies (BioLegend). Live Dump^−^ B cells (CD38^+^), GC B cells (RBD^+^ and GCB RBD^−^), and PCs were then isolated by flow cytometry and loaded onto Chromium Controller from 10X Genomics. Single-cell RNA-sequencing (scRNA-seq) libraries were prepared using Chromium Single Cell 5′ version 2 Reagent Kit (10X Genomics) according to the manufacturer’s protocol. Libraries were loaded onto an Illumina NovaSeq for single-cell gene expression (GEX), VDJ analysis, and hashtag oligo library at the Rockefeller University Genomics Resource Center. Hashtag indexing from TotalSeq-C antibodies was used to demultiplex the sequencing data and generate gene and barcode matrices, respectively.

All single-cell BCR libraries were mapped to the Cell Ranger VDJ GRCm38 reference using Cell Ranger vdj version 8.1.0 (10X Genomics). Contigs containing <50 reads and >1 heavy or light chain were removed. Antibodies heavy and light chains were paired using in-house scripts, and further analyzed using igpipeline version 3 (https://github.com/stratust/igpipeline/tree/igpipeline3) to define clonotypes, as previously described (Wang et al., 2023), using the mouse IMGT database as a reference (Lefranc, 2011).

For the primary response dataset analyses (Fig. 1 H and Fig. 3), scRNA-seq and hashtag oligo unique molecular identifier quantification were performed with Cell Ranger counts version 8.0.1 using the Cell Ranger GEX reference mm10, and analyzed in R with Seurat version 5.1.0 (Hao et al., 2021). Cells were demultiplexed with MULTISeqDemux, and those classified as doublets or with mitochondrial content >10% and feature count <1,000 or >6,500 were excluded. Cell cycle genes were regressed out. Sample batches were then merged, scaled, and normalized with SCTransform. B cell (*Cd19*, *Ms4a1*, *Cd79a*, *Tnfrsf13c*, *Aicda*, *Lyz2*, *Cd63*), follicular (*Cd38*, *Cd55*, *Bcl2*, *Maml2*, *Notch2*, *Gpr183*, *Sell*, *Ccr6*), and memory B cell (Binet et al., 2024, *Preprint*) signatures were assigned using AddModuleScore. Cells with low B cell module score (≤25th percentile) or with high memory B cell score (≥75th percentile) were excluded. B cells expressing *Bcl6*, *Aicda*, *S1pr2*, or *Fas* at high levels (≥75th percentile) were classified as GC B cells, whereas those with high follicular score (≥75th percentile) were classified as follicular B cells.

### Statistical analysis

Details of statistics including tests used, exact values, and *n* numbers are indicated in figure legends and/or main text. Quantification and statistical analyses were performed in R (version 4.4.0) with RStudio server (2024.04.0 Build 735) and/or GraphPad Prism (version 10.2.3), unless otherwise detailed in this Materials and methods section. Graphs generated using Prism and R were assembled into figures using Adobe Illustrator. Flow cytometric analysis was performed in FlowJo version 10.10.0 software (BD).

### Online supplemental material

Fig. S1 contains flow cytometric gating strategies and quantifications, as well as Iglv usage related to Fig. 1. Fig. S2 contains flow cytometric gating and quantification, as well individual mouse clonality data, Vh nucleotide mutations, and Iglv gene usage related to Fig. 3. Fig. S3 depicts ELISA data and VH + VL mutation rate related to Fig. 4. Fig. S4 shows ELISA data and flow cytometric quantifications related to Fig. 4. Fig. S5 contains flow cytometric quantifications, VH + VL mutations, and Iglv gene usage related to Fig. 5. Table S1 contains recombinant antibody sequences and ELISA data. Table S2 lists flow cytometric reagents.

## Supplementary Material

Table S1 shows recombinant antibody sequences and binding data related to Fig. 4.

Table S2 shows flow cytometric reagents.

## Data Availability

All data needed to evaluate the conclusions in the paper are available in the main text and supplementary materials. 10X Genomics datasets generated for this project are publicly available and are archived in the National Center for Biotechnology Information (NCBI) Gene Expression Omnibus (GEO) database under accession number GSE326797. Part of the data for Fig. 3 and Fig. S2 are accessible at NCBI GEO accession number GSE287123. All codes used in this work are publicly available as of the date of publication. Reagents, including mouse strains, are available from H. Hartweger and M.C. Nussenzweig under a material transfer agreement with The Rockefeller University under reasonable request.
